# Efficacy of Modified Qingre Jiedu Decoction Combined with Three-Dimensional Conformal Radiotherapy in Treating Moderate to Advanced Ovarian Carcinoma and Its Effect on Levels of Serum Carcinoembryonic Antigen and Carbohydrate Antigen 125

**DOI:** 10.1155/2022/1821719

**Published:** 2022-06-15

**Authors:** Shufen Ai, Jin Xie

**Affiliations:** ^1^Department of Gynecology, Changqing District People's Hospital of Jinan, Jinan City 250300, Shandong Province, China; ^2^Medical Imaging Center, Central Hospital Affiliated to Shandong First Medical University, No. 105, Jiefang Road, Jinan City, Shandong 250013, China

## Abstract

**Objective:**

To explore the efficacy of modified Qingre Jiedu decoction combined with three-dimensional conformal radiotherapy (3D-CRT) in treating moderate to advanced ovarian carcinoma (OC) and its effect on patients' serum carcinoembryonic antigen (CEA) and carbohydrate antigen 125 (CA125).

**Methods:**

The clinical data of 84 patients with moderate to advanced OC treated in the gynecology department of *Changqing District People's Hospital of Jinan* from February 2017 to February 2018 were selected for retrospective analysis, and the patients were divided into the single chemotherapy group (taxol + carboplatin, *n* = 42) and the combined group (modified Qingre Jiedu decoction+3D-CRT, *n* = 42) according to the parity of their admission numbers. By measuring their levels of serum CEA and CA125 after treatment, the cellular immune levels of the two groups were compared.

**Results:**

Compared with the single chemotherapy group after treatment, the combined group obtained significantly higher total clinical effective rate and 1-year, 2-year, and 3-year survival rates (*P* < 0.05), significantly higher CD4^+^/CD8^+^ and NK cell level values (*P* < 0.001), significantly lower serum CA125 and CEA level values (*P* < 0.001), and significantly lower total incidence rates of toxic and side effects (*P* < 0.05).

**Conclusion:**

The abovementioned results show that the combined treatment modality has a significant effect on prolonging the survival of patients with moderate to advanced OC and can effectively reduce the levels of tumor markers and improve the body's immunity. Further study will be conducive to establishing a better solution for OC patients.

## 1. Introduction

Ovarian carcinoma (OC) is a malignant tumor that occurs in the ovary, which has diverse histologic types and is mainly of epithelial origin. As a gynecological malignant tumor with high mortality, patients who suffer from the disease only have a 5-year survival rate of about 30% [1, 2]. Due to the insidious onset and lack of typical clinical manifestations at an early stage of the tumor, most patients have entered the middle to late stages when diagnosed, and without timely treatment or processing, they will suffer from different degrees of complications, such as tumor rupture, twisting of the pedicle, and severe infections, endangering their life and health. Cytoreductive surgery plus postoperative systemic chemotherapy is currently the main therapeutic modality for patients with moderate to advanced OC [3], which will improve the short-term efficacy of patients, but has a high recurrence rate caused by resistance to chemotherapy, with more than 70% of patients relapsing within 1-2 years. Therefore, how to prolong the survival time and reduce the recurrence rate of such patients has been a challenge for gynecologic oncologists [4].

With the development of imaging and linear accelerator technology, adjuvant radiotherapy has been widely used in treating gynecological diseases. It has been documented that [5] extracorporeal conformal radiotherapy is a common radiation treatment for a variety of tumor diseases, and three-dimensional conformal radiotherapy (3D-CRT) is currently the most frequently used one, which can reduce the irradiated dose to the surrounding organs, reduce the damage to the surrounding tissues, and achieve the effect of precise radiation therapy while ensuring efficacy [6, 7]. Traditional Chinese medicine (TCM) is experienced in treating gynecological diseases. The modified Qingre Jiedu decoction is prepared based on the famous antitumor formula “Lichong decoction” and has a proven antitumor effect. It contains peach seed, dandelion, tuckahoe, Mongolian milkvetch root, and other herbs and can exert efficacy such as promoting blood circulation and detoxication, invigorating the spleen, and replenishing qi [6]. Relevant clinical reports in recent years have shown [8] that the combination of TCM and Western medicine has significant advantages in improving the efficacy and reducing the mortality rate of patients with gynecological tumors. Currently, few reports focus on the efficacy of combining the two, and the results of the combination therapy were reported as follows.

## 2. Data and Methods

### 2.1. General Information

**Figure 1:**
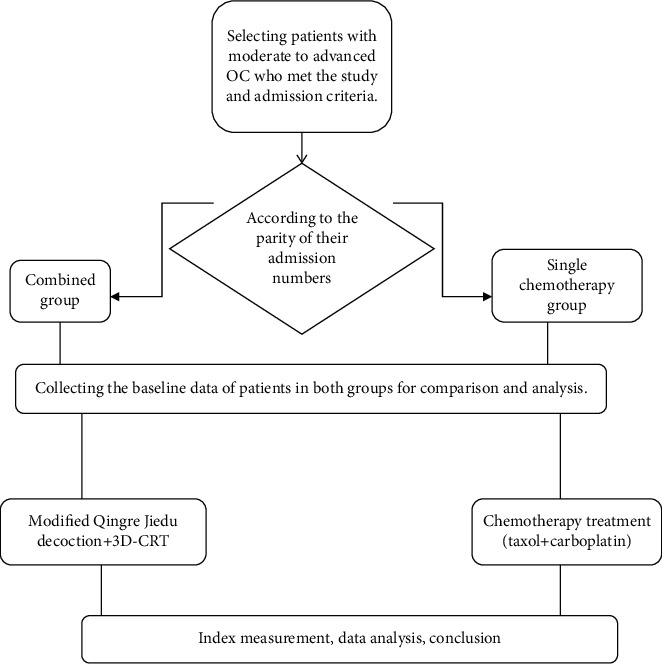
Study technical process.

The clinical data of 84 patients with moderate to advanced OC treated in the gynecology department of *Changqing District People's Hospital of Jinan* from February 2017 to February 2018 were selected for retrospective analysis, and the patients were divided into the single chemotherapy group and the combined group according to the parity of their admission numbers, with 42 cases each. See Figure 1 for the specific study process.

### 2.2. Inclusion and Exclusion Criteria

The inclusion criteria were as follows: ① the patients who met the diagnosis criteria for OC in the 4^th^ edition of *Guidelines for the Diagnosis and Management of Ovarian Malignancies* [7] and were diagnosed after histopathological diagnosis, and the clinical manifestations included lower abdominal distending pain, abdominal mass, and hesitancy in urination; ② their KPS scores were not less than 60 points, and the estimated survival was over 6 months; ③ they met the indications of 3D-CRT; and ④ the study met the *World Medical Association Declaration of Helsinki (2013*) [8] and was approved by the Ethics Committee of *Changqing District People's Hospital of Jinan.*

The exclusion criteria were as follows: ① the patients who had other malignant tumors; ② the patients who suffered from serious cardiovascular and cerebrovascular diseases; ③ the patients who were at least 65 years old or under the age of 18; and ④ the patients who had diseases in the immune system, blood system, nervous system, etc., were allergic to the drugs used in the study, or had poor compliance.

### 2.3. Methods

Taxol + carboplatin therapy was performed on patients in the single chemotherapy group with the following specific steps: 135 mg/m^2^ of taxol (manufactured: Beijing Union Pharmaceutical Factory Co., Ltd.; NMPA approval no. H20083786; specification: 10 ml: 60 mg) was dissolved in 500 mL of normal saline for intravenous drip, 1 h after that, 200 mg/m^2^ of carboplatin (manufactured: Yangtze River Pharmaceutical (Group) Co., Ltd.; NMPA approval no. H20044616; specification: 100 mg) was added to normal saline and administered via intravenous drip within 2 h [9, 10]. Before chemotherapy, it was necessary to use antiallergic drugs and prepare first aid equipment to ensure the safety of patients. The chemotherapy was conducted once every four weeks as one course, with 6 consecutive courses totally.

The modified Qingre Jiedu decoction combined with 3D-CRT treatment was carried out on patients in the combined group.

The formula of the modified Qingre Jiedu decoction was 28 g of tuckahoe, 30 g of largehead atractylodes rhizome, 30 g of dandelion, 8 g of liquorice root, 15 g of oriental water plantain tuber, 15 g of safflower, 10 g of peony root, 10 g of peach seed, 10 g of heterophylly false starwort root, 10 g of Mongolian milkvetch root, and 10 g of Sichuan lovage rhizome. The decoction was decoted with the TCM decoction machine (manufactured: Zhengzhou Xinyao Mechanical Equipment Co., Ltd.; model: XY-BC1 + 1W). After boiling, it was extracted and filtered through the solution machine and packed with the microcomputer packaging machine according to the patients' oral dose, with 200 ml per pack. The patients took 1 pack in the morning and in the evening for 6 consecutive weeks. Patients in both groups accepted regular clinical follow-up visits.

3D-CRT treatment: contrast-enhanced computed tomography (CECT) was adopted for sequential scanning positioning, 5-mm slice thickness scanning, and delineating gross tumor volume and expanding by 0.5–1.0 cm as the planning target area. Four cophase radiation fields were selected and surrounded by a 90% isodose curve, and the optimal treatment regimen was evaluated by dose volume histograms. The external irradiation dose of the linear accelerator four-field box 3D-CRT was 45 Gy, and the afterload treatment was the intracavitary dose of 25–30 Gy.

### 2.4. Observation Indicators

The clinical efficacy after treatment of all patients was evaluated by the response evaluation criteria in solid tumors (RECICT 1.1) [11]. Complete response (CR): disappearance of lesion for over one month; partial response (PR): ≥50% decrease volume of measurable lesion; stable disease (SD): <50% decrease volume of measurable lesion and <25% enlarged lesion; and progression disease (PR): ≥25% increase volume of measurable lesion or new lesions. The objective remission rate (ORR) = CR + PR.

After treatment, 5 ml of fasting elbow venous blood was drawn from patients of both groups to take the serum after anticoagulation and centrifugation. The cellular immune levels, including the level values of CD4^+^, CD8^+^, and natural killer (NK) cells, were measured with the flow cytometer (manufactured: Shanghai 3V Medical Equipment Co., Ltd.; model: FACSVia), and the level values of CD4^+^/CD8^+^ were calculated.

The serum CEA and CA125 level values were measured by enzyme linked immunosorbent assay (ELISA) with the kits purchased from Shanghai Jingkang Bioengineering Co., Ltd., and the operation was carried out in strict accordance with the specifications on the kits.

By 3 years of follow-up, the postoperative 1-year, 2-year, and 3-year survival rates and the incidence rates of toxic and side effects (including gastrointestinal reactions, neutropenia, radiodermatitis, renal dysfunction, and leukopenia) after treatment were compared between the two groups.

### 2.5. Statistical Methods

In this study, the data processing was conducted by the professional statistical software SPSS 23.0, the picture drawing software was GraphPad Prism 7 (GraphPad Software, San Diego, USA), the enumeration data were examined by the *X*^2^ test and expressed by (*n*(%)), the measurement data were examined by the *t*-test and expressed by mean ± SD, and differences were considered statistically significant at *P* < 0.05.

## 3. Results

### 3.1. Comparison of Baseline Data between the Two Groups

The clinical data such as mean age, BMI value, pathological type, and degree of differentiation were not significantly different between the two groups (*P* > 0.05) (Table 1).

### 3.2. Comparison of Clinical Efficacy between the Two Groups

The total clinical effective rate of the combined group was significantly higher than that of the single chemotherapy group (*P* < 0.05). See Table 2.

### 3.3. Comparison of Cellular Immune Level after Treatment between the Two Groups

**Figure 2:**
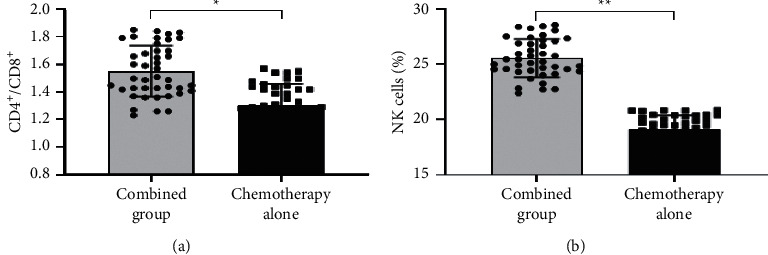
Comparison of cellular immune levels after treatment between the two groups (mean ± SD, *n* = 42). (a) Comparison of the CD4^+^/CD8^+^ level values after treatment between the two groups. The horizontal axis indicates the combined group and the single chemotherapy group, and the vertical axis indicates the values. The mean CD4^+^/CD8^+^ level values of the combined group and the single chemotherapy group after treatment were 1.55 ± 0.19 and 1.30 ± 0.16, respectively. ^*∗*^ indicates a significant difference in the mean CD4^+^/CD8^+^ level values after treatment between the two groups (*t* = 6.523, *P* < 0.001). (b) The comparison of the NK cell level values after treatment between the two groups. The horizontal axis indicates the combined group and the single chemotherapy group, and the vertical axis indicates the values in %. The mean NK cell level values of the combined group and the single chemotherapy group after treatment were 25.56 ± 1.72% and 19.13 ± 1.27%, respectively. ^*∗∗*^ indicates a significant difference in the mean NK cell level values after treatment between the two groups (*t* = 19.490, *P* < 0.001).

After treatment, the level values of CD4^+^/CD8^+^ and NK cells were significantly higher in the combined group than in the single chemotherapy group (*P* < 0.001) (see Figure 2).

### 3.4. Comparison of Level Values of Serum Tumor Markers between the Two Groups

**Figure 3:**
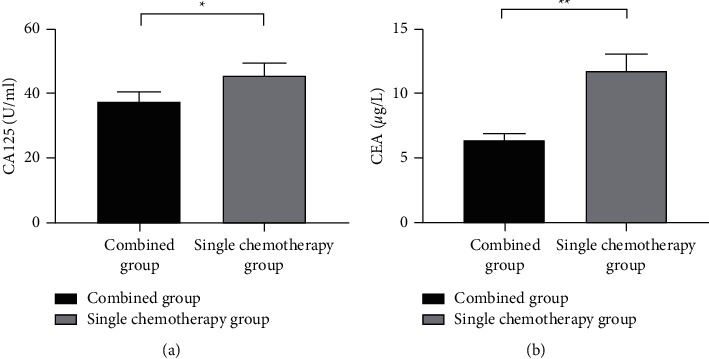
Comparison of level values of serum tumor markers between the two groups (mean ± SD, *n* = 42). (a) The comparison of the CA125 level values after treatment between the two groups. The horizontal axis indicates the combined group and the single chemotherapy group, and the vertical axis indicates the values in U/ml. After treatment, the CA125 level values of the combined group and the single radiotherapy group were 37.39 ± 3.07 and 45.34 ± 3.07, respectively. ^*∗*^ indicates a significant difference in CA125 level values after treatment between the two groups (*t* = 11.867, *P* < 0.001). (b) The comparison of the CEA level values after treatment between the two groups. The horizontal axis indicates the combined group and the single chemotherapy group, and the vertical axis indicates the values in *μ*g/L. After treatment, the CEA level values of the combined group and the single radiotherapy group were 6.31 ± 0.58 and 11.74 ± 1.24, respectively. ^*∗∗*^ indicates a significant difference in CEA level values after treatment between the two groups (*t* = 25.706, *P* < 0.001).

After treatment, the CA125 and CEA level values of the combined group were significantly lower than those of the single chemotherapy group (*P* < 0.001) (Figure 3).

### 3.5. Comparison of Survival Rates at Different Moments between the Two Groups

The 1-year, 2-year, and 3-year survival rates of the combined group were significantly higher than those of the single radiotherapy group (*P* < 0.05) (Table 3).

### 3.6. Comparison of Toxic and Side Effects between the Two Groups

The total incidence rate of toxic and side effects was significantly lower in the combined group than in the single chemotherapy group (*P* < 0.05) (Table 4).

## 4. Discussion

In recent years, the increasing work pressure on women has led to more and more new OC cases every year and shows a younger trend of the disease, which has a mortality rate up to more than 65% [12], ranking the first in female reproductive malignancies. OC is often overlooked for its insidious onset at an early stage, so most patients are at the middle to late stage when they visit the clinic. Survey data have shown [13] that about 75% of patients who present to the hospital with lesions that have involved one or both ovaries are accompanied by abdominal metastasis, with clinical manifestations such as abnormal pain, menstrual disorder, and abnormal vaginal bleeding. In advanced stages, the disease can involve the thoracic cavity, liver, kidney, and other organs and show cachexia signs such as obvious ascites, edema of the lower extremities, and anemia, which are life-threatening. At present, cytoreductive surgery or chemoradiotherapy is mainly adopted for treatment, but clinical investigations have found [14–16] that tumor cells in patients treated in these ways can still rapidly spread into the blood and lymphatic system, leading to a higher recurrence rate. With the stronger toxic and side effects of chemoradiotherapy, most patients will have vomiting, diarrhea, and other intestinal reactions, and some will even have liver and kidney injury, and cytoreductive surgery can only reduce the tumor volume but does not have the ability of radically curing the tumor, which affects their prognosis and rehabilitation [14, 17]. In this study, according to the summary of long-term clinical experience, the modified Qingre Jiedu formula was applied on the basis of 3D-CRT for treating patients with moderate to late OC.

Relevant studies have confirmed [15] that radical radiotherapy has the same effect as surgery. As a radiotherapy technique applied in the clinic for a long time, conformal radiotherapy can reduce the irradiation dose to the surrounding tissue by adjusting the morphology of the irradiated X-ray [16, 18, 19]. Computer and imaging technology are continuously developing, and as an advanced technology of modern radiotherapy, 3D-CRT is based on the principle of controlling the strength of the subrays in each beam, which then controls the dosage distribution, improving the tumor control rate and reducing the damage to normal tissues and organs to the greatest extent. However, 3D-CRT results in significant radiation to normal tissue outside the irradiated area, in addition to universal and equal intensity irradiation within the irradiated area [20]. With the rapid development of TCM, its application jointly with chemoradiotherapy in treating tumors has gradually become a research hotspot. TCM holds that [21] OC belongs to the category of “amassment and accumulation,” which causes severe loss of vital qi, blood flow and fluid, and deficiency of both yin and yang, and then leads to the formation of coagulated phlegm, blood stasis, and internal stagnation, which may easily result in tumor recurrence if not promptly eliminated. Modified Qingre Jiedu decoction has efficacy in clearing heat and eliminating toxins, removing stasis, supporting healthy energy, and invigorating qi and nourishing yin, and among its formula, Mongolian milkvetch root can invigorate qi and spleen, induce diuresis for removing edema, and so on; tuckahoe has the effects of diuresis and diffusing dampness, invigorating the spleen and regulating the stomach, and tranquilizing the mind, and therefore it can enhance body immunity, resist tumors, and protect the liver; Sichuan lovage rhizome can promote blood circulation to remove blood stasis, relieve pain, and reduce swelling; peony root has the function of dissipating blood stasis and relieving pain as well as clearing heat and cooling blood. In modern pharmacology, this formula can accelerate the apoptosis of tumor cells and promote the secretion of immune factors, and its therapeutic efficacy has been demonstrated in advanced cervical cancer [22].

CA125, a glycoprotein detected from OC antigen, is mainly derived from the coelomic epithelium in the embryonic development period and is mostly highly expressed in the serum of OC patients [23]. CEA is a broad-spectrum tumor marker, with a level elevated in the middle to late stages of OC [24, 25]. In this study, the results proved that after treatment, the serum CA125 and CEA levels were significantly lower in the combined group than in the single chemotherapy group (*P* < 0.001), indicating that the combined treatment could effectively kill the tumor cells and reduce tumor burden. In addition, the study also found that the survival rates were significantly better in the combined group than in the single chemotherapy group, indicating that the combined treatment could greatly prolong the survival time of patients with moderate to advanced OC.

The innovations of this study are as follows: ① the comparative analysis between the modified Qingre Jiedu decoction plus 3D-CRT and single chemotherapy was conducted to explore the feasibility of the combination therapy; ② by comparing the total clinical effective rate, survival rate, and toxic and side effects, the advantages of combined treatment in treating moderate to advanced OC were discussed, the application value and clinical meaning of the combined treatment for OC were generally evaluated, and the prospect of clinical application was revealed. The limitations of this study are as follows: ① due to the limited clinical conditions and other factors, only 84 research objects were enrolled in the study, and they were all treated in the local hospitals, so the source of cases lacked diversification; ② only the chemotherapy regimen of taxol plus carboplatin was adopted, and the comparative study with other regimens was lacking; therefore, the completeness of clinical data requires further support from multicenter and large-sample studies. Further confirming that the combined application of modified Qingre Jiedu decoction and 3D-CRT can create a new field for the treatment of OC.

## Figures and Tables

**Table 1:** Comparison of baseline data between the two groups (*n* = 42).

Item	Single chemotherapy group	Combined group	*X* ^2^/*t*	*P*
Mean age (mean ± SD, years)	47.42 ± 4.17	47.48 ± 4.03	0.067	0.947
BMI (mean ± SD, kg/m^2^)	21.26 ± 0.83	21.30 ± 0.76	0.230	0.818
Pathological type				
** **Serous cystadenocarcinoma	26 (61.90%)	24 (57.14%)	0.198	0.657
** **Clear cell carcinoma	12 (28.57%)	10 (23.81%)	0.398	0.528
** **Hybrid cystadenocarcinoma	4 (9.52%)	8 (19.05%)	1.556	0.212
Tumor stage			0.233	0.629
** **III	29 (69.05%)	31 (73.81%)		
** **IV	13 (30.95%)	11 (26.19%)		
Degree of differentiation				
** **Poor differentiation	6 (14.29%)	7 (16.67%)	0.091	0.763
** **Moderate differentiation	26 (61.90%)	27 (64.29%)	0.051	0.821
** **Well differentiation	10 (23.81%)	8 (19.05%)	0.283	0.595
Marital status				
** **Married	33 (78.57%)	35 (83.33%)	0.309	0.578
** **Unmarried	5 (11.90%)	2 (4.76%)	1.403	0.236
** **Divorced	4 (9.52%)	5 (11.90%)	0.124	0.724
Educational degree				
** **College	9 (21.43%)	11 (26.19%)	0.263	0.608
** **High school	25 (59.52%)	21 (50.00%)	0.769	0.381
** **Primary school	8 (19.05%)	10 (23.81%)	0.283	0.595
Place of residence			0.429	0.513
** **Urban area	19 (45.24%)	22 (52.38%)		
** **Rural area	23 (54.76%)	20 (47.62%)		

**Table 2:** Comparison of clinical efficacy between the two groups (*n*(%)).

Group	*n*	CR	PR	SD	PD	ORR
Combined group	42	22 (52.38)	14 (33.33)	4 (9.52)	2 (4.76)	85.71% (36/42)
Single chemotherapy group	42	16 (38.10)	11 (26.19)	9 (21.43)	6 (14.29)	64.29% (27/42)
*X* ^2^						4.200
*P*						<0.05

**Table 3:** Comparison of survival rates between the two groups (*n*(%)).

Group	1-year survival rate	2-year survival rate	3-year survival rate
Combined group	38 (90.48%)	32 (76.19%)	26 (61.90%)
Single chemotherapy group	30 (71.43%)	23 (54.76%)	14 (33.33%)
*X* ^2^	4.941	4.266	6.873
*P*	<0.05	<0.05	<0.05

**Table 4:** Comparison of toxic and side effects between the two groups.

Group	Gastrointestinal reaction	Neutropenia	Radiodermatitis	Renal dysfunction	Leukopenia	Total incidence rate
Combined group	2 (4.76)	1 (2.38)	1 (2.38)	1 (2.38)	2 (4.76)	16.67% (7/42)
Single radiotherapy group	5 (11.90)	3 (7.14)	0 (0.00)	3 (7.14)	4 (9.52)	35.71% (15/42)
*X* ^2^						3.941
*P*						<0.05

## Data Availability

Data used to support the findings of this study are available on reasonable request from the corresponding author.
